# Scapula dyskinesis in medium‐sized full‐thickness rotator cuff tear after subacromial Lidocaine infiltration and rotator cuff reconstruction

**DOI:** 10.1002/jeo2.70154

**Published:** 2025-02-06

**Authors:** Zoltán Nyőgér, Csenge A. Molnár, Norbert Szakály, Anna Várnagy, Tamás Terebessy, Tibor Gunther, Gábor Skaliczki

**Affiliations:** ^1^ Department of Health and Sport Sciences Szechenyi István University Győr Hungary; ^2^ Department of Mechatronics, Optics and Mechanical Engineering Informatics, Faculty of Mechanical Engineering Budapest University of Technology and Economics Budapest Hungary; ^3^ Department of Orthopaedics Semmelweis University Budapest Hungary

**Keywords:** rotator cuff repair, scapula dyskinesis, shoulder joint biomechanics, subacromial injection, vicon motion capturing system

## Abstract

**Purpose:**

In rotator cuff tears, scapular dyskinesis is often observed. The aim of our study is to better understand the cause and the role of scapular dyskinesis in rotator cuff tears and evaluate changes in scapulothoracic kinematics after Lidocaine subacromial injection and surgery in patients with medium‐sized (1–3 cm) rotator cuff tear.

**Methods:**

The scapular motion during humerus sagittal flexion of nine healthy persons (healthy group, HG) and nine persons with a medium‐sized rotator cuff tear (surgery group, SG) was investigated using the VICON motion capture system and upper limb evaluation in movement analysis software. In addition, quality of life and functional outcomes were assessed in the SG group using American Shoulder and Elbow Surgeons, Oxford and Constant‐Murley scores and rotator muscle force and Visual Analogue Scale score were evaluated. The SG was further divided into three subgroups: measurements were performed preoperatively (before surgery native subgroup—BSN), then after subacromial Lidocaine injection (before surgery injection subgroup—BSI) and 6 months after rotator cuff reconstruction (after surgery subgroup—AS). Changes observed after injection (BSI) and surgery (AS) were compared to the BSN.

**Results:**

In the BSI, a significant reduction (*p* < .025) in protraction was observed in the raising phase between 20° and 70° comparing it to the BSN, protraction decreased by 5.3° ± 7.9° (mean ± standard deviation [SD]). In the lowering phase between 80° and 30°, we registered a decrease of protraction by 6.0° ± 8.3° (mean ± SD). In the AS, we observed an approximation of protraction to the HG, but no significant change was detected.

**Conclusion:**

Significant reduction in scapular protraction was demonstrated with Lidocaine subacromial injection during both the arm raising and lowering phases. Six months of rehabilitation treatment in the postoperative period is not enough to fully eliminate scapular dyskinesis.

**Level of Evidence:**

Level II.

**Abbreviations**
AS: after surgery subgroup
ASES: American Shoulder and Elbow Surgeons
BSI: before surgery injection subgroup
BSN: before surgery native subgroup
CM: Constant‐Murley
HG: healthy group
IQR: interquartile range
N: Newton
n.s.: not significant
RC: rotator cuff
SD: standard deviation
SG: surgical group
ULEMA: upper limb evaluation in movement analysis
VAS: Visual Analogue Scale

## INTRODUCTION

Rotator cuff (RC) tears can lead to serious shoulder complaints at any age, usually resulting in shoulder pain, reduced range of motion, impaired scapular motion and abnormal shoulder muscle function [8, 11, 16, 25, 27, 33]. In case of an RC tear, lifting of the arm can still be possible through a coordinated action of the scapulothoracic and glenohumeral joints; however, the centralizing effect of the RC becomes insufficient and an abnormal scapular movement pattern, called scapula dyskinesis, occurs showing the disturbed kinetic chain [10, 12, 18, 19, 22, 30].

A number of studies have investigated movements of the shoulder girdle, particularly the scapula [25, 29, 32]. In healthy humans, the predominant movement pattern of the scapula during elevation is upward rotation in the scapular plane, external rotation in the horizontal plane and posterior tilting in the sagittal plane relative to the thoracic‐based coordinate system. However, for shoulder abnormalities, such as RC pathologies, no clear pattern of movement in either direction or the extent of scapular motion has been described [7, 20, 29, 30, 32]. Some studies have suggested that changes in scapular motion are primarily due to pain and impingement syndrome [21, 35], while other studies have directly attributed scapular dyskinesis to RC tears [32, 37]. In clinical practice, it can be seen in many cases that patients with moderate RC tears can often use their shoulders without pain with very good function.

The primary aim of our study was to investigate the scapulothoracic kinematics after subacromial Lidocaine injection and surgery. We hypothesized that RC tears play a role mainly through pain in the development of scapular dyskinesis in medium‐sized RC tears; if the pain is eliminated, the abnormal movement pattern of the scapula improves. We also assumed that dyskinesis resolves after 6 months of rehabilitation following successful RC reconstruction. The second aim of our study was to investigate clinical outcomes such as RC muscle strength, arm lift changes and pain in the before surgery injection subgroup (BSI) and after surgery subgroup‐(AS) compared to the before surgery native subgroup (BSN).

## MATERIAL AND METHOD

### Participants, surgical procedure

Our prospective, monocentric study was conducted between September 2020 and September 2022 at the Department of Orthopaedics, Semmelweis University, Budapest, Hungary. Subjects were divided into two groups. The healthy group (HG) consisted of nine voluntary subjects, who had never had a shoulder complaint, shoulder surgery or injury. This group was used solely for scapulothoracic kinematic analysis. An ultrasound (Samsung HS 60, 16 MHz linear head) examination was performed in all cases to rule out any possible RC and AC joint pathology. Yamaguchi suggests that in cases of torn RC, a significant percentage of patients also have RC pathology on the opposite shoulder, and, therefore, we chose healthy subjects as the control group and did not use the other shoulder in the surgical group (SG) as a control [38].

The SG included nine subjects, who had magnetic resonance imaging (MRI)‐confirmed medium‐sized (1–3 cm) full‐thickness RC tears with persistent pain despite conservative therapy (physiotherapy, non‐steroidal anti‐inflammatory drugs) and, therefore, were scheduled for RC repair. MRI scan showed Goutallier I in seven patients and Goutallier II in two patients. Exclusion criteria for our study were cervical radiculopathy, glenohumeral instability, previous shoulder fracture, trauma, muscular dystrophy, previous stroke, AC arthritis, rheumatoid arthritis and previous shoulder surgery. The demographics of the groups are summarized in Table 1.

**Table 1:** Patient demographic data.

	HG	SG	*p* Value
Number of patients	9	9	‐
Male/female	7/2	6/3	‐
Age (mean ± SD)	57.6 ± 9.8	59.2 ± 7.8	n.s.
Dominant side right/left	7/2	7/2	‐
Affected side right/left	7/2	8/1	‐
BMI (mean ± SD)	28.4 ± 3.4	28.7 ± 3.0	n.s.

*Note*: Age and BMI values of HG and SG subgroups were compared. Two‐sample t‐test was used to calculate the *p* value.

Abbreviations: BMI, body mass index; HG, healthy group; n.s., not significant; SD, standard deviation; SG, surgery group.

All surgical procedures were performed by the same surgeon (G. S.) using a combination of general anaesthesia and interscalene blockade in beach chair position. Arthroscopic (Arthrex Synergy 4K) single‐row reconstruction technique was used with one or two 5.5 mm Corkscrew FT III anchors (Arthrex). Given this size of RC tear, there is no difference in the results when considering single‐row, double‐row and transosseous‐equivalent techniques [6, 28]. The arm was immobilized in a sling for 6 weeks postoperatively. Passive and assisted active range of motion exercises guided by a physiotherapist were started on the first postoperative day. After 6 weeks, active movements and muscle‐strengthening exercises were started, also supervised by a physiotherapist. The time between diagnosis and surgery was 87.3 ± 20.9 days.

### Outcome collection

The examinations were performed in a sitting position. All subjects underwent the same evaluation protocol. We asked the participants to lift their arms in the sagittal plane starting from their side to the maximum height they could reach. The sagittal flexion was measured three times, and the average of the measurements was used for the calculations. During the sagittal flexion of the humerus, the rotational movements of the scapula were also tested three times for each participant and their mean values were used for the calculations. The primary purpose of this measurement was to evaluate scapula rotations. In the meantime, the maximum sagittal flexion of the humerus was also assessed.

Members of both groups (HG and SG) were subjected to motion analysis by the VICON motion capture system (Nexus 2.10, Oxford Metrics). The patients in the SG were assessed on three occasions:

1. Before surgery, without receiving any medication (BSN).
2. Before surgery, 10 min after landmarked‐based administration of 10 mL of 1% Lidocaine solution (EGIS, Hungary) into their subacromial space (BSI).
3. Six months after surgery (after surgery subgroup—AS).

Patients of the SG received regular physiotherapy after surgery, and an ultrasound (Samsung HS 60, 16 MHz linear head) was performed before the final measurement at 6 months to check the reconstructed RC.

### Clinical outcome collection

In the SG group, the quality of life and functional outcomes were measured by Constant‐Murley score, American Shoulder and Elbow Score and OXFORD Shoulder score [4]. The rotator muscle strength was measured using a dynamometer. As we mentioned earlier, the humerus sagittal flexion was analyzed by VICON. Pain level was measured using a Visual Analogue Scale (VAS).

Muscle strength was measured in every subgroup (SG consist of BSN, BSI and AS) during the Jobe test, the bear‐hug test and by rotation in next‐to‐the‐body position using a dynamometer (SDF‐300, China). The strength tests were measured three times, and the average of the measurements was used for the calculations.

### Movement analysis system

**Figure 1:**
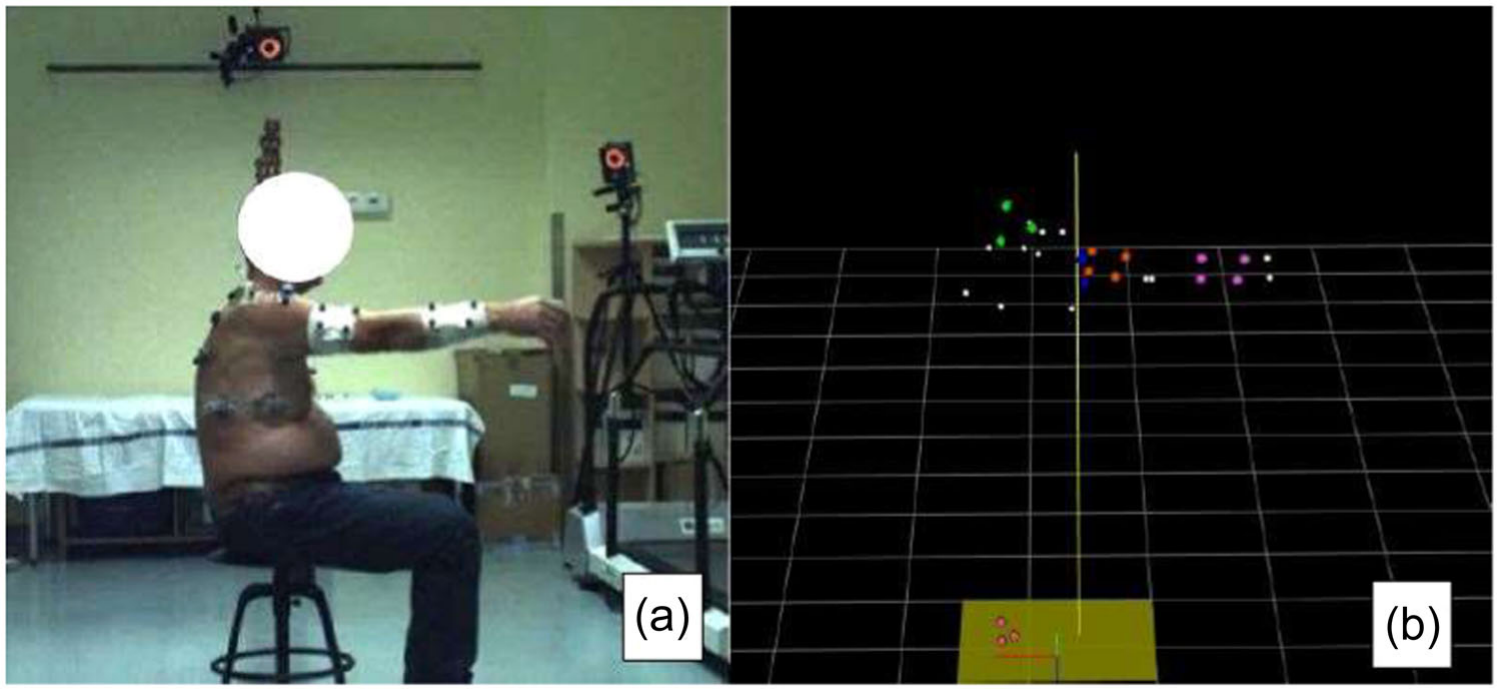
VICON and upper limb evaluation in movement analysis (ULEMA) markers. (a) The live image showing all the actual markers detected by the VICON system. (b) The version displayed by VICON and ULEMA with all the actual (coloured) and calibration markers (white), 30 markers in total.

We used a VICON motion capture system (Nexus 2.10, Oxford Metrics) equipped with seven infrared cameras, six of which are MX T40 and one of which is a separate Vantage five camera. In addition to the infrared cameras, the system also includes two cameras that record the actual footage of the measurement. In collaboration with the VICON system, we used the upper limb evaluation in movement analysis (ULEMA) software, ver. 1.2.1 (2020), which performs its calculations using MATLAB (MATLAB (R2020a), The MathWorks Inc., Natick, Massachusetts, United States) [9]. Working in the infrared range, the cameras detect a total of 17 physical markers fixed at predefined points on the subjects' bodies. In addition to the physical markers, 13 virtual markers were also recorded by the ULEMA software using a dedicated calibration stick. The system is capable of analyzing the three‐dimensional (3D) kinematics of the upper limb, using both physical and virtual markers. During exercises, the change in the position of the physical markers is detected by VICON, while the changes in the position of the virtual markers are calculated by the ULEMA algorithms. Thus, a total of 30 points are recorded for each measurement, 17 physical and 13 virtual markers (Figure 1).

The accuracy of the VICON system for tracking markers is <0.15 mm for static measurements and up to 0.30 mm for dynamic measurements [23]. The kinematic calculations for the upper limb are based on the rigid body model assumption, with local coordinate systems assigned to each segment (trunk, scapula, humerus, forearm, hand). The software uses ISB guidelines for the calculation of anatomical coordinate systems and joint angles [36].

### Data processing

**Figure 2:**
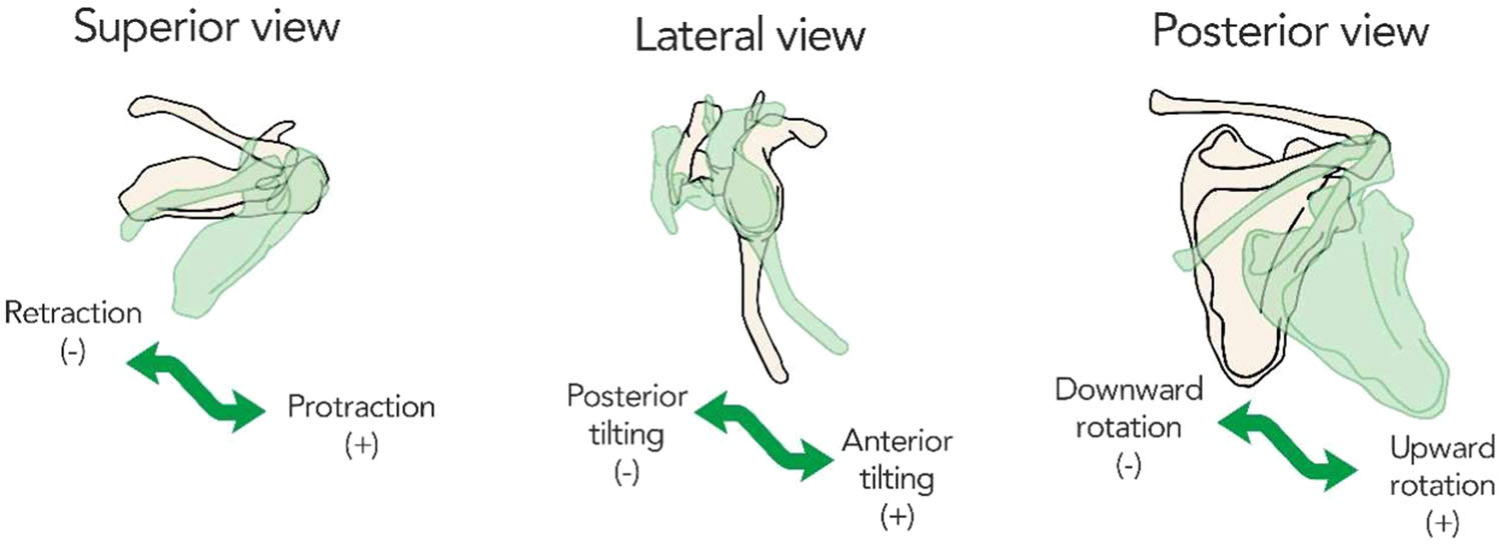
Scapula rotations with display of directions.

The following 3D kinematic parameters of the shoulder girdle were analyzed: humerus sagittal flexion/extension (+/−), scapula protraction/retraction (+/−), scapula anterior tilting/posterior tilting (+/−) and scapula upward rotation/downward rotation (+/−) (Figure 2). The scapular motion was studied during the sagittal flexion of the humerus. Patients in the BSN could raise their arms to an average of 114°, so scapular movements during arm elevation were evaluated between 20° and 110°. In humerus sagittal flexion, we analyzed the values of scapular rotations every 10° in each group, subgroup (HG, BSN, BSI, AS).

### Statistical analysis

Before the statistical analysis, the normality of all data was assessed with the Lilliefors test. In the case of scapular protraction/retraction, anterior/posterior tilting and upward/downward rotation at every 10° of humeral sagittal flexion, a significant part of the data did not pass the normality test. To handle all the cases uniformly, and due to the presence of outliers, Wilcoxon's signed rank test was applied to compare the scapular angles of BSN–BSI and BSN–AS. Functional scores and quality of life outcomes, muscle strength, maximal humeral sagittal flexion and VAS score of the three analyzed subgroups successfully passed the normality test; therefore, paired *t* test was used to discover differences between muscle strength, maximal humeral sagittal flexion and VAS score of BSN‐BSI and BSN–AS. Bonferroni correction was used for all statistical tests, therefore, level of statistical significance was set at *p* < .025. Calculations were performed using MATLAB R2020b. Continuous variables are expressed as mean ± standard deviation.

## RESULTS

The ultrasound in patients who have undergone surgery showed an intact RC in all cases after 6 months.

### Scapular movements

**Figure 3:**
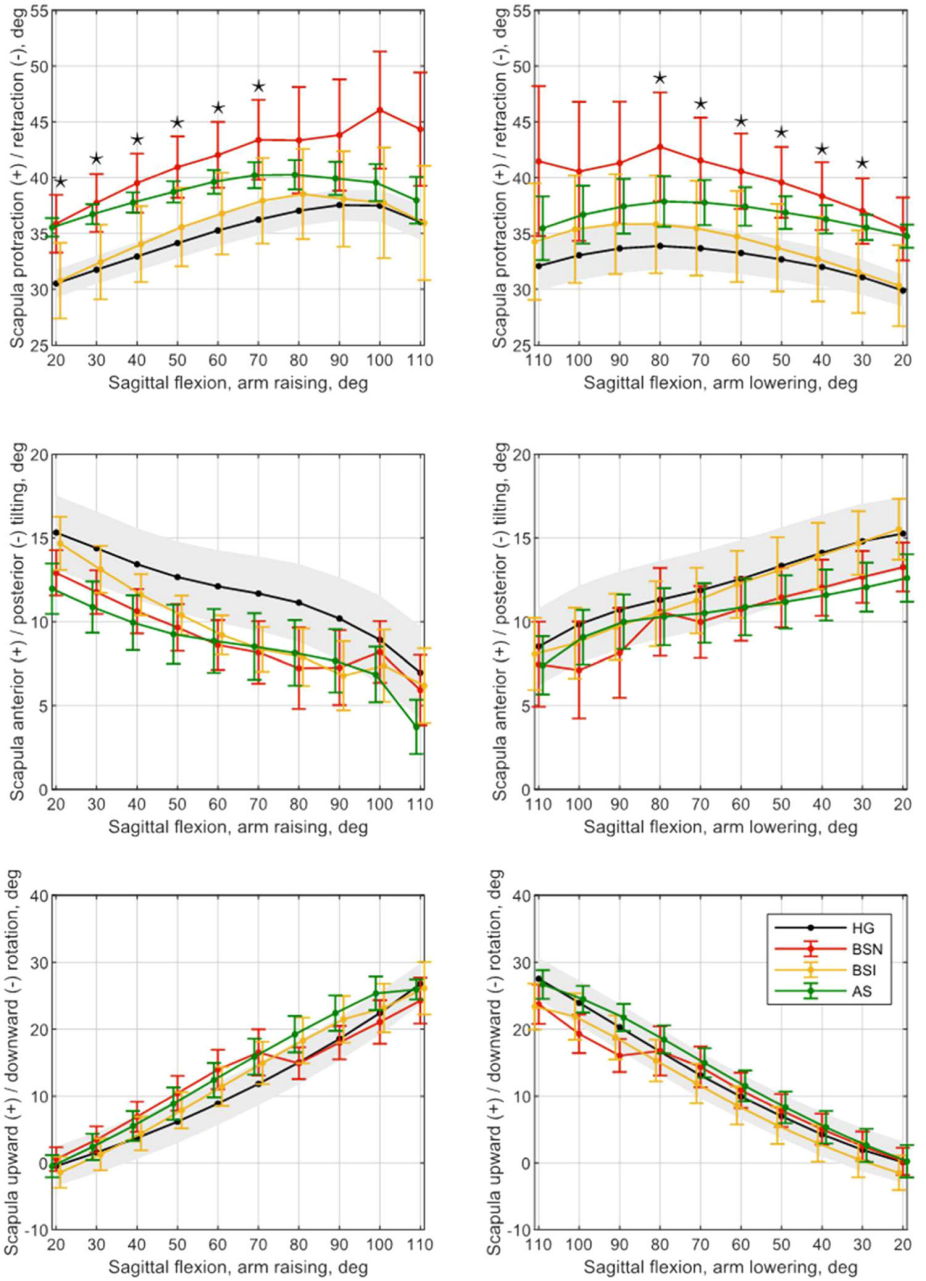
Mean curves of angular values of scapular rotations during raising and lowering of the arm in the sagittal plane. Black, red, yellow and green colour stand for healthy group (HG), before surgery native subgroup (BSN), before surgery injection subgroup (BSI) and after surgery subgroup subgroups, respectively. Colourful error bars show the standard error of the corresponding group/subgroup. The upper and lower bands of the grey‐shaded area indicate the standard error of the HG group. Statistically significant difference (*p* < 0.025) between BSN and BSIsubgroups is denoted by *. Matlab R2020b and Inkscape were used to create the figure.

In our previous work, we compared scapular rotation values during humerus sagittal flexion between the HG and BSN groups, both during arm raising and lowering [25]. Increased protraction has been detected in the BSN both in the raising and lowering phases but the change did not reach the level of significance. No significant difference in anterior/posterior tilting and upward/downward rotation movements was confirmed in either the raising or lowering phases. The results are shown in Figure 3, with HG in black line and a band of the grey‐shaded area indicating the standard error of the HG group.

In our current work, we compared the scapular movements of the SG group between the different subgroups (BSN and BSI, BSN and AS) during arm raising and lowering. Comparing the BSN and BSI, a significant difference in protraction was observed throughout the raising phase between 20° and 70° (Table 2), the average decrease of protraction is 5.3° in the BSI and the mean of the standard deviation is 7.9° (Table 3). Also, in the lowering phase, a significant difference was measured in protraction between 80° and 30° (Table 2), resulting in an average decrease of 6.0°; the mean of the standard deviation is 8.3° (Table 3), (Figure 3). No significant difference in anterior/posterior tilting and upward/downward rotation movements was confirmed in either the raising or lowering phases (Table 2).

**Table 2:** Statistical results of deviations in scapula rotations during arm raising and lowering after injection and after surgery.

Arm raising							Arm lowering						
	BSN–BSI			BSN–AS				BSN–BSI			BSN–AS		
dgr	scap. proret. *p* value	scap. a/p tilting *p* value	scap. up/dn rot. *p* value	scap. proret. *p* value	scap. a/p tilting *p* value	scap. up/dn rot. *p* value	dgr	scap. proret. *p* value	scap. a/p tilting *p* value	scap. up/dn rot. *p* value	scap. proret. *p* value	scap. a/p tilting *p* value	scap. up/dn rot. *p* value
20ᵒ	.020*	n.s.	n.s.	n.s.	n.s.	n.s.	110ᵒ	n.s.	n.s.	n.s.	n.s.	n.s.	n.s.
30ᵒ	.004*	n.s.	n.s.	n.s.	n.s.	n.s.	100ᵒ	n.s.	n.s.	n.s.	n.s.	n.s.	n.s.
40ᵒ	.004*	n.s.	n.s.	n.s.	n.s.	n.s.	90ᵒ	n.s.	n.s.	n.s.	n.s.	n.s.	n.s.
50ᵒ	.008*	n.s.	n.s.	n.s.	n.s.	n.s.	80ᵒ	.008*	n.s.	n.s.	n.s.	n.s.	n.s.
60ᵒ	.008*	n.s.	n.s.	n.s.	n.s.	n.s.	70ᵒ	.008*	n.s.	n.s.	n.s.	n.s.	n.s.
70ᵒ	.016*	n.s.	n.s.	n.s.	n.s.	n.s.	60ᵒ	.012*	n.s.	n.s.	n.s.	n.s.	n.s.
80ᵒ	n.s.	n.s.	n.s.	n.s.	n.s.	n.s.	50ᵒ	.020*	n.s.	n.s.	n.s.	n.s.	n.s.
90ᵒ	n.s.	n.s.	n.s.	n.s.	n.s.	n.s.	40ᵒ	.020*	n.s.	n.s.	n.s.	n.s.	n.s.
100ᵒ	n.s.	n.s.	n.s.	n.s.	n.s.	n.s.	30ᵒ	.020*	n.s.	n.s.	n.s.	n.s.	n.s.
110ᵒ	n.s.	n.s.	n.s.	n.s.	n.s.	n.s.	20ᵒ	n.s.	n.s.	n.s.	n.s.	n.s.	n.s.

*Note*: Significant differences (*p* < .025) are marked with *.

Abbreviations: AS, after surgery subgroup; BSI, before surgery injection subgroup; BSN, before surgery native subgroup; dgr, degree; n.s., not significant; scap. a/p tilting, scapula anterior/posterior tilting; scap. proret., scapula protraction/retraction; scap. up/dn rot., scapula up/down rotation.

**Table 3:** Difference between BSN–BSI scapula protraction.

BSN–BSI comparison													
	Protraction arm raising							Protraction arm lowering					
dgr	Mean change	Median change	SD	IQR	CI 95%		dgr	Mean change	Median change	SD	IQR	CI 95%	
20ᵒ	−5.1*	−1.1*	8.4	6.5	**−**11.6	1.4	110ᵒ	**−**2.3	**−**2.0	1.7	2.7	**−**4.4	**−**0.2
30ᵒ	−5.3*	−1.7*	8.1	5.8	**−**11.5	1.0	100ᵒ	**−**5.4	**−**3.1	7.2	3.6	**−**13.0	2.1
40ᵒ	−5.5*	−2.0*	8.0	5.5	**−**11.6	0.7	90ᵒ	**−**5.6	**−**3.4	7.2	3.3	**−**13.1	2.0
50ᵒ	−5.4*	−2.1*	7.7	5.8	**−**11.3	0.6	80ᵒ	−7.2*	−3.7*	8.4	9.2	**−**14.3	**−**0.2
60ᵒ	−5.2*	−2.0*	7.6	5.9	**−**11.4	0.6	70ᵒ	−6.1*	−2.9*	8.4	5.6	**−**12.6	0.4
70ᵒ	−5.7*	−2.0*	8.0	8.0	**−**12.4	1.0	60ᵒ	−5.9*	−2.6*	8.2	4.9	**−**12.2	0.5
80ᵒ	**−**3.9	**−**1.9	6.0	1.0	**−**10.2	2.4	50ᵒ	−5.9*	−2.8*	8.3	5.1	**−**12.2	0.5
90ᵒ	**−**4.2	**−**2.2	6.2	0.5	**−**10.7	2.3	40ᵒ	−5.6*	−2.6*	8.4	5.2	**−**12.1	0.8
100ᵒ	**−**1.9	**−**2.2	0.7	0.7	**−**2.8	**−**0.9	30ᵒ	−5.4*	−1.9*	8.5	5.9	**−**12.0	1.1
110ᵒ	**−**1.5	**−**1.6	0.7	0.8	**−**2.3	**−**0.6	20ᵒ	**−**5.1	**−**1.1	8.7	7.1	**−**11.8	1.6

*Note*: Significant differences (*p* < 0.025) are marked with *. *p* values are given in Table 2.

Abbreviations: BSI, before surgery injection subgroup; BSN, before surgery native subgroup; CI, confidence interval; dgr., humerus sagittal flexion degree; IQR, interquartile range; SD, standard deviation.

The protraction movement curve of AS impacts the HG both in the lifting and lowering phase; consequently, a normalization of the movement pattern can be seen, but the change did not reach the level of significance (Figure 3).

### Functional scores and quality of life outcomes

Comparing the results of the BSN and AS (Table 4), we found a significant improvement in the AS for all three scores.

**Table 4:** Comparison of Oxford, ASES, CM scores and muscle strength, humerus maximum sagittal flexion and VAS between BSN, BSI and AS.

	BSN	BSI	AS	*p* value
OXFORD score	27.7 ± 10.2		43.5 ± 3.5	≤.0001*
ASES score	46.7 ± 19.3		86.5 ± 10.1	≤.0001*
CM score	48.2 ± 17.3		77.2 ± 7.5	≤.0001*
Jobe test (*N*)	32.0 ± 18.2	32.9 ± 19.1	47.6 ± 20.0	BSN–BSI, n.s.
				BSN–AS, n.s.
Bear‐hug test (*N*)	64.9 ± 16.9	67.19 ± 16.9	96.5 ± 36.0	BSN–BSI, .0136*
				BSN–AS, .0042*
External rotation force (*N*)	34.2 ± 11.1	36.0 ± 9.3	50.2 ± 17.8	BSN–BSI, n.s.
				BSN–AS, n.s.
Humerus maximum sagittal flexion (dgr)	114.7° ± 33.5°	132.4° ± 21.6°	144.4° ± 18.6°	BSN–BSI, .0163*
				BSN–AS, .0144*
VAS score	5.5 ± 1.2	2.7 ± 0.7	2.1 ± 0.8	BSN–BSI, ≤.0001*
				BSN–AS, ≤.0001*

*Note*: Significant differences (*p* < .025) are marked with *. Data of score, muscle strength, maximum sagittal flexion and VAS are given as mean ± SD.

Abbreviations: AS, after surgery subgroup; ASES, American Shoulder and Elbow Score; BSI, before surgery injection subgroup; BSN, before surgery native subgroup; CM, Constant‐Murley; dgr, degree; N, Newton; n.s., not significant; SD, standard deviation; VAS, Visual Analogue Scale.

### RC forces

No significant improvement was observed in the Jobe and external rotation strength test between BSN and BSI, BSN and AS. However, we observed a significant muscle strength increase in the bear‐hug test in both BSI and AS (Table 4) comparing it to that of the BSN.

### Maximum sagittal flexion

Significant change was detected in the maximum flexion after administration of the injection, maximum flexion increased by 17.77° ± 17.01° in the BSI. Also, significant improvement was detected in the AS (Table 4), with an increase of 29.77° ± 28.05° comparing it to that of the BSN.

### Pain

Examining the VAS before (BSN) and after injection (BSI) and 6 months AS, significant pain reduction was found in both groups by injection 2.89 ± 0.93, by surgery 3.44 ± 1.13, respectively (Table 4).

## DISCUSSION

In our study, we observed a significant decrease in scapular protraction after Lidocaine subacromial injection. We also observed a reduction after surgery in scapular protraction, but this did not reach a significant level. After both injection and surgery, there was a significant reduction in pain, a significant increase in muscle strength in the bear‐hug test and an improvement in the maximum sagittal flexion of the humerus. Functional scores and quality of life outcomes also showed significant improvement 6 months after surgery.

We hypothesized that RC tears mainly play a role in the development of scapular dyskinesis through pain in medium‐sized RC tears. In our previous work, we have shown that in moderate RC tears, a difference in protraction is mainly observed (Figure 3) [25]. Kolk and colleagues also studied the surgical outcomes of moderate RC tears [14]. A significant difference was also observed in scapular protraction compared to the control group. Also, the most significant change was observed in this scapular motion as a result of surgery.

In our work, significant pain reduction on the VAS scale was measured after Lidocaine injection. A significant increase in the degree of maximal elevation during sagittal flexion and a significant improvement in bear‐hug muscle strength were confirmed in the BSI (Table 4). Ettinger investigated the effect of subacromial injection in patients with impingement syndrome during arm scapular plane flexion, where he found increased anterior tilting at flexion above 90° and increased scapular upward rotation between 60° and 90° [5]. Our results are partially in concordance with those of Ettinger's as we also observed increased anterior tilting during sagittal flexion in almost the entire lifting and lowering cycle. In addition, a clear tendency toward a return to physiological movement pattern in protraction was observed with pain reduction (Figure 3). Scibek also investigated the effect of subacromial injection in patients with RC tears [30]. Similar to our observations, in their sagittal flexion tests, they also observed a significant difference in protraction‐retraction movements. Our results showed that by reducing pain in patients with moderate RC tears, scapular protraction affects the healthy individuals both in the raising and lowering phases with a concomitant increase in muscle strength. This may suggest that the abnormal scapular motion is not only caused by biomechanical factors but mostly by pain. These results also support our hypothesis.

The review of the literature shows that in large RC tears, a significant increase in upward rotation is mainly observed in torn RC [2, 15, 24, 26]. Based on our own data and literature, we consider it possible that scapular dyskinesis may have a pain‐avoiding role in moderate tears, while in large tears it may contribute to arm elevation through increased upward rotation. It seems that in moderate tears, the RC is still compensated and able to perform its function. However, in large tears, the RC is no longer able to perform its function and that dyskinesis may be the result of a compensatory process whereby upward rotation of the scapula can reduce the loss of motion of the shoulder joint. Consequently, scapular dyskinesis may be an adaptive process in medium‐sized tears.

A further part of this study evaluated the effect of pain and rehabilitation on dyskinesis development. We also hypothesized that if the pain is eliminated, the abnormal movement pattern of the scapula improves, and that dyskinesis resolves after 6 months of rehabilitation following successful RC reconstruction. There are many protocols for the postoperative management of RC tears, which may differ in the duration of fixation, initiation of passive and active exercises, accelerated or conservative [3]. Nevertheless, comparative studies show that there is no meaningful difference between patients' range of motion and satisfaction at 6 and 12–24 months [1, 17]. Therefore, we chose to perform postoperative reassessment at 6 months.

In our research in the AS group, 6 months after operation, changes in scapular rotation were observed in all three rotation forms, with protraction showing a trend toward the HG, consequently, the pattern of movement was closer to the HG (Figure 3). Kolk examined his patients 1 year after reconstruction of moderate RC tears and found significant differences in scapular protraction compared with that of the preoperative status [14]. In the current study, we also demonstrated a decrease in protraction in the postoperative period, but the difference was not statistically significant. It is possible to expect improvement in scapular dyskinesis beyond 6 months. Song investigated moderate to large supraspinatus and infraspinatus tears [31]. Scapular rotations during arm sagittal flexion were analyzed 1 year after surgery, with improvement in scapular dyskinesis noted in 52.1% of patients. The most significant improvement in posterior tilting was observed in 75% of patients. We could not identify a clear change in posterior tilting and upward rotation in the AS but found an improvement in protraction toward the HG movement pattern. This may be due to the difference in the size of the tears (we investigated medium‐sized tears) and the difference in the follow‐up time (6 months in our study). Scapulothoracic compensation may result in an increased upward rotation in large RC tears, while medium tears may have less upward rotation. Ueda investigated scapula upward rotation during humerus scapular plane elevation in small and massive RC tears at 2 and 5 months after surgery [34]. He observed significant differences in both RC tear groups preoperatively compared with the control group. The different scapula upward rotation remained significant even 5 months after surgery in both groups comparing it with the control group; however, the group with a small tear demonstrated a significant decrease in scapula upward rotation at 120° of arm elevation compared with that of the preoperative status. In the massive tear group, there was no significant improvement observed. In contrast, in our study, no differences in scapula upward rotation values were found in the comparison with BSN–BSI, BSN–AS. Our results may be explained by the fact that in the case of medium‐sized tears there is no need for increased upward rotation of the scapulothoracic junction, because the glenohumeral joint movements can still be performed with the help of the moderately torn RC.

Our current study shows that in moderate RC tears the improvement trends of scapula dyskinesis in the BSI and AS are toward to the HG. But the normal scapular motion pattern does not return fully after 6 months of postoperative rehabilitation. Our results suggest that pain is primarily responsible for scapular dyskinesis in medium‐sized RC tears. These results raise the possibility that the main direction of dyskinesis in medium‐sized tears would be increased protraction, while in large tears it would be increased upward rotation of the scapula.

Further studies are needed to determine the main dyskinesis directions related to the size of the tears (small, medium, large, massive). Accurate knowledge of these may help both conservative therapy without surgery and postoperative physiotherapy.

## LIMITATIONS

There are a few limitations to our investigation. First, the number of patients is relatively low, which could bias our results and underpower the statistical analysis. However, motion analysis studies generally investigate small study groups due to the nature of these intensive investigations [13, 34]. On the other hand, despite the small number of elements, the *p* values are quite low in the protraction values where significant differences could be demonstrated. Thus, with an increase in the number of patients, no change in the reporting of the main results is expected, possibly a quantitative change, but not a qualitative one. Second, in the healthy control group, only an ultrasound scan of the RC was performed. Third, we did not measure the tear intraoperatively.

On the other hand, several factors support the strength of our study. First, our measurements were compared to a HG with demographic characteristics similar to the study group. Second, the members of the study group had homogeneous tear sizes, so we were able to examine similar conditions. Third, the effects of injection and surgery were studied in the same group. Fourth, we measured scapular rotations in both the raising and lowering phases of the arm, bringing the measurements closer to the clinical setting, where we often find that scapular dyskinesis also occurs in the lowering phase [8].

## CONCLUSION

Based on our study, in isolated medium‐sized RC tears pain plays an important role in the development of scapular dyskinesis. It appears that scapular dyskinesis in these cases is more of a consequence of a compensatory mechanism to avoid pain than that of a biomechanical cause. As the pain is reduced, the scapular protraction decreases and approaches the healthy pattern.

Finally, we found that despite continuous physiotherapy, scapular dyskinesis was observed 6 months after arthroscopic reconstruction of isolated medium‐sized RC tears.

## AUTHOR CONTRIBUTIONS

The study was designed by Gábor Skaliczki, Zoltán Nyőgér and Anna Várnagy. Literature research was conducted by Zoltán Nyőgér, Tamás Terebessy and Tibor Gunther. The patients were operated on by Gábor Skaliczki. The measurements were completed by Zoltán Nyőgér, Norbert Szakály and Anna Várnagy. Statistical calculations were carried out by Csenge A. Molnár. The article was written by Zoltán Nyőgér, Gábor Skaliczki and Csenge A. Molnár. All authors read and approved the final manuscript.

## CONFLICT OF INTEREST STATEMENT

The authors declare no conflicts of interest.

## ETHICS STATEMENT

Our work was approved by the Regional, Institutional Scientific and Research Ethics Committee SE RKEB 90/2020. All subjects gave written informed consent. Informed consent was obtained from all individual participants in this study.

## Data Availability

The data that support the findings of this study are available from the corresponding author upon reasonable request.
